# Pedigree and Molecular Analyses in the Assessment of Genetic Variability of the Polish Greyhound

**DOI:** 10.3390/ani11020353

**Published:** 2021-01-31

**Authors:** Małgorzata Goleman, Ireneusz Balicki, Anna Radko, Iwona Rozempolska-Rucińska, Grzegorz Zięba

**Affiliations:** 1 Department of Ethology and Wildlife Management, University of Life Sciences, Akademicka 13, 20-950 Lublin, Poland; 2 Department and Clinic of Animal Surgery, University of Life Sciences, Głęboka 30, 20-612 Lublin, Poland; balicki.ireneusz@gmail.com; 3 Department of Animal Molecular Biology, National Research Institute of Animal Production, Krakowska 1, 32-083 Balice, Poland; anna.radko@izoo.krakow.pl; 4 Institute of Biological Basis of Animal Production, University of Life Sciences, Akademicka 13, 20-950 Lublin, Poland; iwona.rucinska@up.lublin.pl (I.R.-R.); grzegorz.zieba@up.lublin.pl (G.Z.)

**Keywords:** Polish Greyhound, pedigree analysis, founders, molecular analysis

## Abstract

**Simple Summary:**

The Polish Greyhound is an old Polish breed. The first descriptions of hunting with greyhounds can be found in the hunting literature of the 16th century, and the first detailed description of a greyhound comes from 1600. The Polish Greyhound probably originates from the Saluki-type Asian greyhounds. The population of this breed is very small and limited mainly to the territory of its country of origin (Poland). Pedigree books were opened for this breed in 1981. The narrow gene pool necessitated mating relatives, resulting in the appearance of various genetic diseases in the breed. The analysis of Polish Greyhound pedigrees registered in the pedigree books from the time of the registration of the breed and molecular tests facilitated calculation of the degree of relatedness in the breed and to design future breeding plans, taking into account that a 2.56% increase in inbreeding per complete generation will be observed if the current breeding program and similar matings are maintained.

**Abstract:**

The aim of the study was to assess the genetic variability of the Polish Greyhound population based on pedigree analysis and molecular DNA testing and to determine the degree of relatedness among individuals in the population. Pedigree data of 912 Polish Greyhounds recorded in pedigree books since they were opened for this breed were analyzed. For molecular testing, DNA was obtained from cheek swabs taken from 235 dogs of the tested breed. A panel of 21 markers (Short Tandem Repeat—STR) was used. The mean inbreeding determined for the Polish Greyhound population based on pedigree analyses was low and amounted to 11.8%, but as many as 872 individuals of the 912 dogs in the studied population were inbred. A total of 83 founders (at least one unknown parent) were identified, among which 27 founders had both unknown parents. Full-sibling groups consisted of 130 individuals, with a minimum and maximum litter size of 2 and 16, respectively. The average litter size was 5.969. Gene diversity calculated based on the mean kinship matrix was 0.862 and the population mean kinship was 0.138. The founder genome equivalent based on the mean kinship matrix was 3.61; the founder genome surviving level was 12.34; the mean Ne was estimated at 21.76; and the Ne/N ratio was 0.135. The FIS inbreeding coefficient for 21 STR was negative, and the mean FIS value for all loci had a low negative value (−0.018). These values suggest a low level of inbreeding in the examined breed as well as the avoidance of mating related animals.

## 1. Introduction

**Figure 1:**
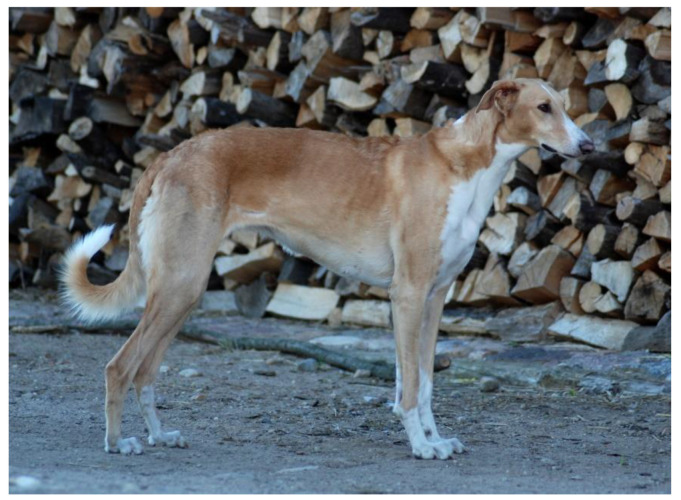
Body of a Polish Greyhound, female: IZIS Akacjana—owner Marta Kościańska (photo: Marta Kościańska).

**Figure 2:**
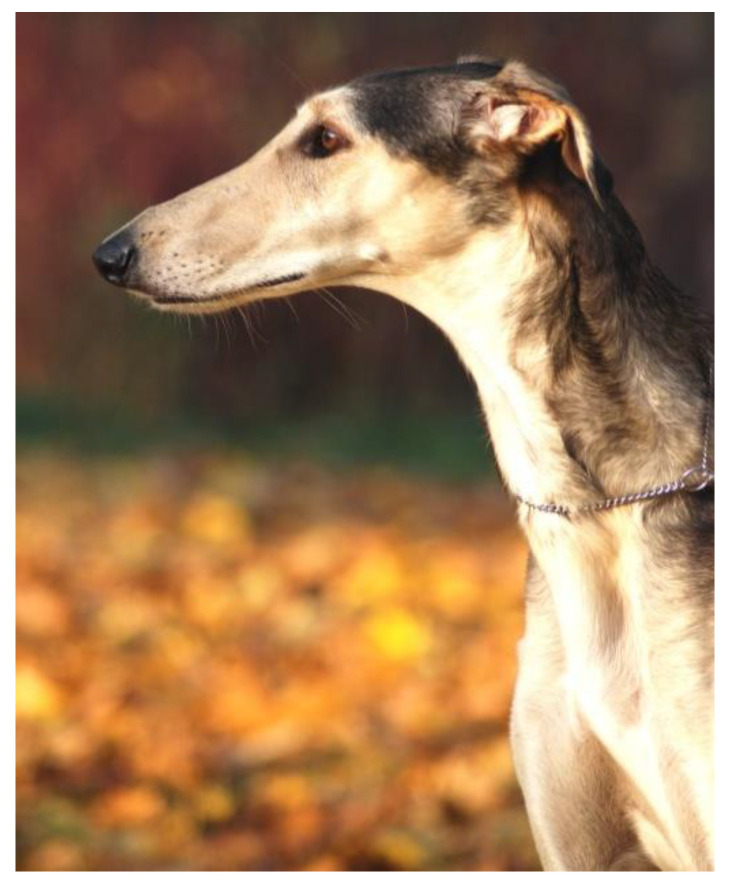
Head of a Polish Greyhound. female: TRZMIELINA Divinacanis, in grizzly color with domino marks—breeder and owner, Marta Kościańska (photo: Marta Kościańska).

The Polish Greyhound is the oldest Polish dog breed. Even the medieval chronicles of Gallus Anonymus report the considerable expenses of the royal court for the maintenance of greyhounds. The first descriptions of hunting with greyhounds can be found in the hunting literature of the 16th century, and the first accurate description of a greyhound at that time dates to 1600. The Polish Greyhound probably comes from the Saluki-type Asian greyhound. It was originally used for hunting fowl (e.g., great bustards). It is a large, muscular, strong dog, clearly stronger than other short-haired sighthounds. Strong bones, compact body, distinct muscles, and strong jaws prove that the dog was used for hunting in the difficult conditions of the Polish climate (Figure 1 and Figure 2). It was very difficult to recreate the breed in the post-war years (World War II), as there were only a few individuals left, which were mostly in the hands of poachers. By a resolution of January 25, 1981, the Plenum of the Main Board of the Polish Kennel Club decided to open the Preliminary Book for the Polish Greyhound. Małgorzata Szmurło was responsible for the care of documentation and breeding development [1]. Based on the literature and iconography, the standard of the Polish Greyhound breed was developed in 1981. It was fully recognized by the *Fédération Cynologique Internationale* (FCI) in 1988, and the breed standard was entered under number 333 [2].

Due to the small number of individuals and a small gene pool, various defects, probably genetically determined, are beginning to appear in this breed. Therefore, the Club of the Polish Greyhound (part of the Polish Kennel Club) began to monitor the breed for diseases such as congenital eye defects or hip dysplasia. Information provided by investigations of the genetic structure of the population such as the effective population size, analysis of the genetic structure, and detection of historical bottlenecks [3] is particularly useful in rare native dog breeds in which the so-called genetic bottleneck is observed. It is most often associated with the use of several popular stud dogs and a small gene pool within a small population. This reduces the genetic diversity of the population and increases the incidence of unfavorable alleles and thus hereditary diseases [4]. 

When properly kept, studbooks for breeding animals provide complete information on a given population and are a useful source of data for analyzing the genetic diversity and structure of a given population. Pedigree books are a key element of breeding work, especially in the case of breeds that are not numerous. The possibility of registering individuals of unknown origin (NN) in pedigree books may help to increase the genetic diversity of such breeds. Molecular tests, which yield valuable information about protection and management of a given species, can help to monitor changes taking place within the studied group of individuals. DNA microsatellite markers (Short Tandem Repeat—STR), which allow direct assessment of the genetic variability of various dog breeds, are the most frequently used molecular markers [5,6,7,8]. By analyzing the frequency distribution of alleles of high-polymorphic microsatellite loci, it is possible to monitor the genetic structure of the population and detect changes in the frequency of genes caused, e.g., by breeding work, which contributes to the maintenance of genetic variability in farm animals. The assessment and monitoring of genetic variability facilitate the selection of the optimal mating partner and determination of the degree of relatedness or inbreeding in the studied population based on the estimated indicators. 

The International Society for Animal Genetics (ISAG) recommends a basic set of 21 STR markers for verification of the dog pedigree, which allows determination of the origin of the indicated parents with 99.9% probability and verification of the pedigree of a given individual [9,10]. 

The aim of the study was to assess the genetic variability of the Polish Greyhound population based on pedigree analysis and molecular DNA testing and to determine the degree of relatedness between individuals in the population.

## 2. Material and Methods

The pedigree data of Polish Greyhounds was provided by the Polish Kennel Club. The data of dogs included in the Polish Pedigree Book (Polska Księga Rodowodowa—PKR) comprises four generations of the ancestors of a given individual. Dogs with incomplete origins (with fewer than 3 ancestors on the father’s and mother’s side) were recorded in the Preliminary Book (Księga Wstępna—KW). 

Pedigree data on 912 Polish Greyhounds (433 males and 479 females) were obtained from both studbooks (PKR and KW), which have compiled the relevant information since their opening for this breed in 1981.

### 2.1. Pedigree Analysis

The data were completed, arranged, and then analyzed using the CFC 1.0 Software computer program [11]. Additionally, the pedigree data were analyzed with the PMx ver. 1.2.20140905 (Zoological Society, Chicago, 2014), assuming that an individual from 1.5 to 10 years of age can have offspring. This assumption was based on the Polish Kennel Club Breeding Regulations, where the stud female can be bred until 8 years old. However, an additional one-time mating of a female aged over 8 years that previously gave birth to valuable offspring can be allowed by the Polish Kennel Club upon request from the breeder. There is no upper breeding age for male stud dogs [12,13]. 

The founders of the breed were distinguished based on pedigree analysis. The inbreeding coefficient (F) was calculated for each individual. The number of inbred individuals, full-sibling groups, and average family size was determined, and the genetic variation in the breed was calculated. Founder genome equivalents, founder genome surviving, and effective population size were calculated as well [14,15]. The breeding statement made by the Polish Kennel Club was used to estimate the number of animals owned by breeders.

DNA for molecular tests was obtained from cheek swabs taken from 235 living dogs of the analyzed breed (126 females and 109 males). The dogs were not selected according to any criteria for genetic material collection (random sample). The samples were collected from Polish Greyhounds whose owners agreed to make dogs available for the research (as part of a research project on congenital vision defects). The swabs were preserved in 96% alcohol immediately after collection. The samples were stored in refrigeration at about −18 °C until the start of the analysis. In total, 174 samples that that were suitable for analysis were tested. Over the 8-year research and sample collection period, the loss of 61 samples was related to storage and transportation. (DNA could not be isolated from these samples.)

### 2.2. DNA Isolation

DNA was extracted from buccal swabs using the Sherlock AX Kit (A&A Biotechnology, Gdynia, Poland)) following the manufacturer’s protocol. The extracts were quantified with a NanoDrop 2000 spectrophotometer (Thermo Scientific, Wilmington, DE, USA).

### 2.3. Selection of Markers

A panel of 21 markers (STR) recommended for parentage in domestic dogs by the International Society for Animal Genetics (ISAG) was used: AHTk211, CXX279, REN169O18, INU055, REN54P11, INRA21, AHT137, REN169D01, AHTh260, AHTk253, INU005, INU030, FH2848, AHT121, FH2054, REN162C04, AHTh171, REN247M23, REN105L03, AHTh130, and REN64E19 (http://www.isag.us/Docs/AppGenCompAnim2014_cor.pdf). The panel is standardized across multiple laboratories for canine genotyping (Table 1).

### 2.4. 21-Plex PCR Amplification of DNA

STR loci were amplified using Qiagen Master Mix reagents. The PCR reaction was performed using a Veriti^®^ Thermal Cycler amplifier (Applied BioSystems, Foster City, CA, USA) in a thermal process: 5 min of DNA initial denaturation at 98 °C followed by 30 cycles of denaturation at 98 °C for 15 s, primer attachment to the matrix at 58 °C for 75 s, primer extension at 72 °C for 30 s, and final primer extension at 72 °C for 5 min.

### 2.5. Electrophoresis on ABI 3130xl

The PCR products were analyzed using an ABI 3130xl capillary sequencer from Applied BioSystems (Foster City, CA, USA). The amplified DNA fragments were subjected to electrophoresis in 7% denaturing POP-7 polyacrylamide gel in the presence of a standard length of 500 Liz and a reference sample. The results of the electrophoretic separation were analyzed automatically using the GeneMapper ^®^ Software 4.0 (Applied BioSystems, Foster City, CA, USA).

### 2.6. Statistical Analysis

To characterize the 21 autosomal STR markers of the analyzed Polish Greyhound population, the following parameters were estimated: polymorphic information content (PIC) [16], observed heterozygosity(Ho), expected heterozygosity(He) [17], and inbreeding coefficient (FIS) [18].

Deviations from the Hardy–Weinberg equilibrium (HWE) were tested with the Markov Chain Monte Carlo exact test [19]. The parameters were as follows: 100 batches and 10,000 dememorization steps.

The statistical analysis was carried out in the IMGSTAT IZ PIB program.

## 3. Results

**Figure 3:**
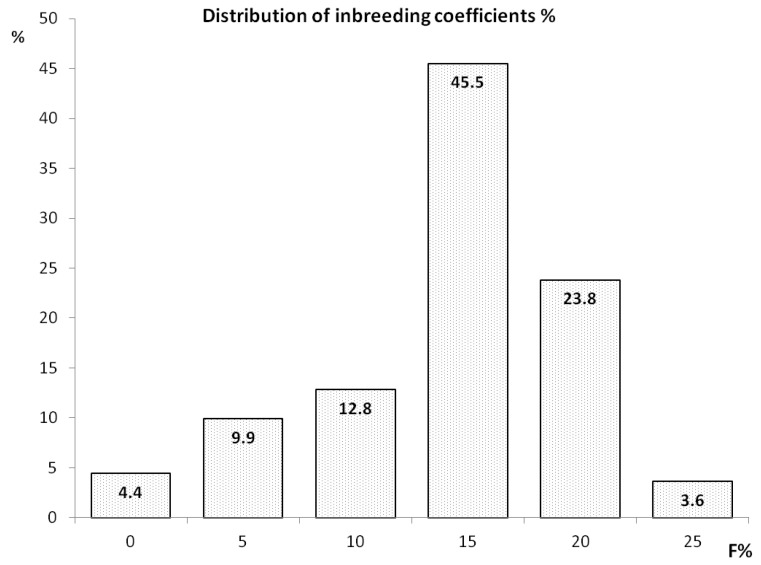
Distribution of inbreeding coefficients (%).

The pedigree analyses showed that 872 individuals in the entire population of 912 Polish Greyhounds were inbred. Mean inbreeding in the population was 11.8% (SD, 5.9%). Furthermore, 23.8% individuals were inbred at a level of over 20%. This value was higher than 25% in 33 individuals. Eight highly inbred dogs had no offspring (Figure 3). Average relatedness exceeding 25% was detected in 38 individuals, with a mean average relatedness of 17.25% in the analyzed population.

The mean pedigree depth covered by the analysis (mean maximum generations) was 6.66, and the mean number of complete generations was 2.95. The intergeneration interval was 5.1 (SE 0.25). The completeness of the pedigree information in the analyzed population is sufficient for the assessment of parameters of genetic variability. 

In the analyzed Polish Greyhound population, 257 (28.18%) with progeny were evaluated based on the pedigrees, including 114 males and 143 females. In total, the females and males had 826 and 829 progeny, respectively. There were 655 dogs with no progeny.

In all, 83 founders (at least one unknown parent) were identified, 27 of which had both unknown parents. The sex ratio of founders was 15 males, which left 51 progeny, to 23 females, which left 92 progeny (indicating the use of 45.78% of the founders in the breeding). There were 45 founders with no offspring.

The number of non-founder dogs numbered 829, including 99 males and 120 females, which were used in breeding. The non-founder males and females left 778 and 734 progeny, respectively, whereas 610 non-founders left no progeny.

The full-sibling groups consisted of 130 individuals, with a minimum litter size of 2 and a maximum size of 16. The average litter size was 5.969.

Two matings were detected between full siblings, which accounted for 0.22%; 21 matings between half siblings, which constituted 2.30%; and two parent-offspring matings, which represented 0.22%.

**Figure 4:**
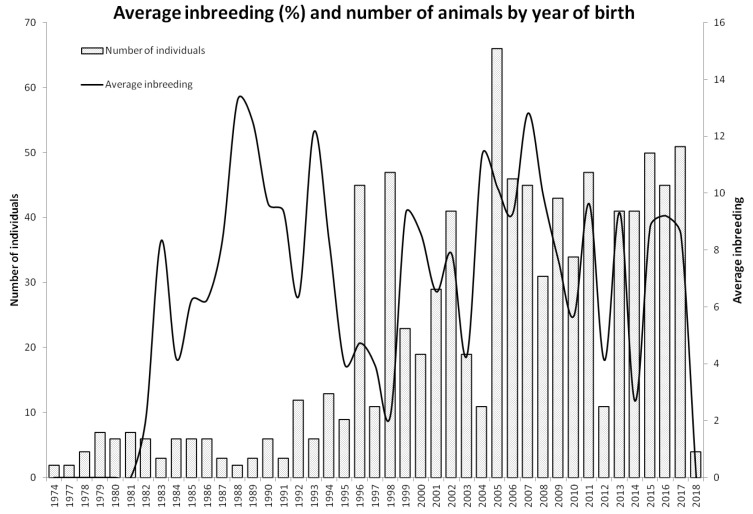
Average inbreeding (%) and number of animals by year of birth.

An indirect picture of the mating history in the Polish Greyhound population may be provided by the number of individuals recorded in the pedigree in each birth year and changes in the level of inbreeding in the analyzed birth years (Figure 4). A small number of individuals in a generation results in a higher inbreeding level. When matings do not result in an increase in homozygosity, greater numbers of individuals in a given birth year contribute to the reduction or maintenance of inbreeding at an unchanged level.

A 2.56% increase in inbreeding per complete generation will be observed if the current breeding program and similar matings are maintained.

The gene diversity calculated based on the mean kinship matrix was 0.862 and the population mean kinship was 0.138. The founder genome equivalent based on the mean kinship matrix was 3.61, the founder genome surviving value was 12.34, the mean Ne was 21.76, and the Ne/N ratio was 0.135.

The molecular analysis based on microsatellite markers showed that all the analyzed microsatellites were polymorphic, with the number of alleles in a locus ranging from 4 in loci AHTk211, INRA21, INU030, REN247M23, and AHT130 to 8 in locus AHT171. Table 2 shows the frequencies of alleles detected in all microsatellite loci in the Polish Greyhound population. 

Based on the frequency of 117 alleles identified in all loci, polymorphisms of 21 microsatellite DNA sequences were determined in the analyzed dog breed. 

The polymorphism was estimated at nearly 60% (average PIC = 0.597). Polymorphism exceeding 60% was observed for 8 STR markers, while a level over 70% was detected for 4 loci (AHT121, FH2054, AHTh171, and REN64E19). The lowest polymorphism (36%) was noted in locus AHTh130. A reduced degree of heterozygosity was also noted for this marker (Ho = 0.37, He = 0.38), while the Ho value for the other loci ranged from 0.43 to 0.82 (Table 2). The mean observed heterozygosity for all STR was 66%.

The FIS inbreeding coefficient for 21 STR was negative, and the mean FIS for all loci had a low negative value (−0.018), which proves the low level of inbreeding as well as systematic avoidance thereof through selection of animals for mating. Positive values for FIS were obtained for nine loci ranging from 0.005 for REN247M23 to 0.07 for INU005 and FH2054.

The Hardy–Weinberg Equilibrium (HWE) assessment carried out with the Markov Chain Monte Carlo exact test [19] showed lack of Hardy–Weinberg equilibrium in six loci at the level of *p* < 0.05 and in five loci at the level of *p* < 0.01.

## 4. Discussion

According to the documentation of the Polish Greyhound Club, it is estimated that there are currently approximately 1000 Polish Greyhounds in Poland. The owners of some of them are not members of the Polish Kennel Club, and these dogs are not intended for breeding. Data from the club show that, according to the latest reports available in 2017–2018, there are 225 registered Polish Greyhounds (96 males and 129 females). Only 64 of them are stud animals (38 males, 26 females). Despite the very small group of dogs of this breed in 2009, an increase in the population was observed when 150 individuals of this breed (70 males and 80 females) were registered, including 52 stud animals (26 males and 26 females) according to the club data. Only 28.18% of the 912 analyzed pedigree dogs registered in the pedigree books were used for breeding. 

Investigations conducted by Lewis et al. [20] have shown that some pedigree dog breeds have high levels of inbreeding and a high burden of inherited diseases unrelated to selection objectives, implying the loss of genetic diversity, which may be a considerable problem in pedigree dogs.

The number of founders with both unknown parents in the Polish Greyhounds was similar to that calculated in the case of Polish Hunting Dogs (27 and 26, respectively), while a fairly large difference in this number was found in all founders with at least one unknown parent (83 individuals in the Polish Greyhounds and only 31 individuals in Polish Hunting Dogs) [21]. Considering the number of potential founders (living individuals at reproductive age that have not had offspring), there were only 4 such Polish Greyhound individuals vs. 22 individuals in the group of Polish Hunting Dogs [21], which indicates the nearly full use of dogs of unknown origin (NN) in breeding. However, the analysis of the use of all founders (with at least one unknown parent) from the beginning of the studbook establishment revealed as many as 45 with no offspring; therefore, the use of the group of founders for breeding purposes was 45.78%. This indicates that many dogs of unknown origin have not been used in breeding, and some of the diversity that these dogs may have introduced into the Polish Greyhound population has been lost. A comparison with two other Polish dog breeds showed that there were 19 founders in Polish Hounds [22] and 44 founders in Polish Tatra Shepherds, but only the Podhale Region was included in the analysis [23]. As reported by Leroy et al. [24] in a study on less-popular French breeds, 49 founders were identified in the Braque Saint-Germain and 13 in the Barbet breeds. In turn, 179 founders were identified in the Istrian Shorthaired Hound by Djurkin Kušec et al. [25]. The average relatedness was estimated to be 17.25% in the Polish Greyhound, while a very low level (1.46%) was reported in the Istrian Shorthaired Hound [25]. As indicated by the comparison of the Polish breeds of hunting dogs, the participation of the Founder Genome Equivalent in the Polish Greyhounds was 3.61, which was lower than in Polish Hunting Dogs (4.17) [21] and higher than in Polish Hounds (1.287) [22]. As shown by Mäki [26], this value was 2.1 in Nova Scotia Duck Tolling Retrievers and 6.4 in Lancashire Heelers. The number of the founder genome equivalent of a population is the same as the number of equally contributing founders with no random loss of founder alleles in descendants [15].

Similarly, the founder genome surviving values in the Polish Greyhounds were lower (12.34) than in Polish Hunting Dogs (17.03). A similarly lower value was achieved by the average effective population size (mean Ne), which was estimated at 21.76 for the Polish Greyhounds and at 28.51 for Polish Hunting Dogs [21]. The gene diversity calculated as part of the analysis of Polish Greyhounds pedigree data was 0.862, which was similar to the value reported in the study on Polish Hunting Dogs (0.880) [21]. The values of both the mean Ne and gene diversity in the Polish Greyhound are comparable to that reported in other rare dog breeds and indicate sufficient variability in the analyzed breed.

The population mean kinship value of the Polish Greyhounds was slightly higher than that of Polish Hunting Dogs reported by Goleman et al. [21] (0.138 and 0.120, respectively). Both these values were clearly lower than those reported by Oliehoek et al. [27] in a small population of Icelandic Sheepdogs (0.23). However, the value for the more popular English Greyhound breed was 0.072, as shown by Calboli et al. [28]. The population mean kinship value in beagles was in the range of 0.010–0.093 [29], but values differ between various dog breeds and are substantially lower in numerous and popular breeds. The mean inbreeding value was similar in the Polish Greyhounds (0.118) and in Polish Hunting Dogs (0.115) [21]. In contrast, the value in a Polish Hound population calculated in 2000–2004 was substantially higher (0.37), as reported by Głażewska [22]. In the Polish Tatra Shepherd from the Tatra Mountain Region, which is another Polish breed (branches of Polish Kennel Club Zakopane and Nowy Targ) investigated by Kania-Gierdziewicz et al. [23], the mean inbreeding value calculated for each sex separately amounted to 0.087 for females and 0.099 for males. In other breeds, it was considerably higher: 0.33 in the Norwegian Lundehund [30], 0.26 in the Icelandic Sheepdog [27], and 0.26 in the Nova Scotia Duck Tolling Retriever [26]. The value reported in Lancashire Heeler dogs by Mäki [26] was similar (0.10) to that in the Polish Greyhounds. The value in popular dog breeds was 0.024 in Labrador Retrievers, 0.073 in Rough Collies [28], and 0.052 in German Dachshunds [31]. Some authors also obtained very low mean inbreeding values in less popular breeds: 0.025 in the Alpine Dachsbracke breed (Alpine short-legged hound) [32], 0.010 in Basset Hound [33], and 0.004 in the Istrian Shorthaired Hound [25].

The Polish Greyhound is a native breed constituting a relatively small population. It is not popular outside of the country of origin. Therefore, the results of the pedigree analyses prove sufficient diversity and heterozygosity in this breed, especially in comparison with findings reported by other researchers.

The calculated average polymorphism degree PIC value based on 174 Polish Greyhound dogs was 0.597, similar to that in the Bracco Italiano, where PIC calculated on the basis of the same 21 STR set was 0.589 [5]. As reported by Botstein et al. [16], markers with PIC values greater than 0.5 are considered to be very informative, values between 0.25 and 0.50 are fairly informative, and values lower than 0.25 are not very informative. Other studies carried out on 18 STR in Polish Tatra Shepherds and 15 STR in German Shepherds showed similar PIC values of 0.598 and 0.558, respectively [6,7]. A higher degree of polymorphism (0.614) calculated on the basis of 15 microsatellite markers was observed in Labrador Retrievers [6]. The PIC index calculated on the basis of 14 STR in 5 dog breeds in South Korea ranged from 0.66 for the German Shepherd to 0.88 for the Jindo [8]. Kang et al. [8] showed high mean polymorphism information content at the level of 0.73 in the Greyhound breed. In comparison with other breeds, the Polish Greyhound is characterized by a moderate degree of polymorphism. This indicates that it exhibits similar genetic variability in the analyzed loci as the other breeds mentioned above.

The highest level of polymorphism was observed in locus AHT171, where 8 alleles were detected and the PIC exceeded 0.7. An equally high degree of polymorphism of this marker was observed in other dog breeds in Poland: the Borzoi (8 alleles, PIC = 0.815), the Tatra Shepherd (8 alleles, PIC = 0.827), and the Polish Hunting Dog (10 alleles, PIC = 0.752) [7,10,21].

The lowest polymorphism was noted for marker AHTh130; one of the 4 alleles identified in this locus, which was 127 bp long, exhibited a markedly higher frequency exceeding 77%, and the PIC value was 0.36. Studies conducted by Ciampolini et al. [5] in American Pit Bull Terrier Dogs and by Goleman et al. [21] in the Polish Hunting Dog indicated high polymorphism of this marker, with PIC values of 0.69 and 0.63, respectively.

At this stage of the research, it is impossible to identify the causes of the limited variability of locus AHTh130 in the Polish Greyhound. A reduced degree of heterozygosity was noted for this marker as well (Ho = 0.37).

The mean observed heterozygosity calculated for all STR was 0.66. A study based on 33 STR in Italian Greyhounds showed slightly lower Ho values of 0.62 and 0.61 in dogs of European origin and from the U.S., respectively [34]. A similar degree of heterozygosity ranging from 0.61 to 0.75 was observed in other dog breeds [7,10,21,35]. The mean observed heterozygosity in the Polish Greyhound breed does not differ from those reported in the case of other breeds.

The ratio of observed to expected heterozygosity was used to calculate the coefficient of inbreeding (FIS), which expresses the degree of inbreeding in a population. Statistically significant FIS values > 0 may indicate inbreeding and an increase in homozygosity related to selection of breeding animals, while FIS values < 0 mean that there is an excess of heterozygotes in the population, which may result from selection favoring heterozygotes. In the study population, the estimated He values were generally similar to the Ho values. The inbreeding coefficient for 12 STR was negative, and the mean FIS value for all 21 loci had a low negative value (−0.018), which suggests no inbreeding in the breed. Similar values were reported by Ciampolini et al. [5] in the Bracco Italiano (FIS = 0.061), Radko et al. [7] in the Tatra Shepherd (FIS = −0.005), Bigi et al. [35] in 6 livestock guard dog breeds (average FIS value = 0.024), and Goleman et al. [21] in the Polish Hunting Dog (FIS = −0.01).

The test for Hardy–Weinberg Equilibrium (HWE) showed significant deviation from genetic equilibrium in Polish Greyhounds. In as many as 5 loci, the absence of Hardy–Weinberg equilibrium was observed at the value *p* < 0.01, whereas the FIS values were rather close to zero. It is possible that the differences in allele frequencies may be an effect of the division of the populations into smaller sub-populations over many generations. In addition, the limited number of dogs in our study may also have caused the deviation of the populations from HWE. Deviation from HWE was observed in other dog populations as well [8,35,36].

## 5. Conclusions

Based on the present results of pedigree analyses, it can be concluded that the Polish Greyhound is characterized by a low inbreeding degree, similar to other native breeds that are not popular. The inbreeding coefficient FIS had a negative value of close to zero in more than half of the assessed loci, which confirms the low level of inbreeding in the studied population. However, there are many individuals related to each other to a different degree. This is inevitable in such a small population of bred animals (at present 64 individuals) and proves correct selection of pairs for mating, avoiding mating of closely related dogs. As in the Polish Hunting Dogs population, the preliminary books for the Polish Greyhound are open and entries of dogs of unknown origin are received by breeders with great enthusiasm. Nowadays, finding a dog of unknown origin (NN) unrelated to such a small breed population is practically impossible. Dogs handled by poachers are also a problem. They come from the FCI breeding but are bred outside the Polish Kennel Club and their offspring are sometimes retrieved and recognized as NN dogs. Without assessment of the relatedness of animals on the basis of information contained in DNA and creation of a pedigree database that will be reliably and systematically replenished, dogs without ancestry recorded in preliminary books may contribute to an increase in homozygosity and reveal inbreeding depression. Pedigree books should include the genetic profiles of individual dogs introduced in breeding, and these data should be available to breeders.

## Figures and Tables

**Table 1:** Primer sequences used for amplification of DNA microsatellite markers.

	STR	F: 5′-3′	R: 5′-3′
1	AHTk211	TTAGCAGCCGAGAAATACGC	ATTCGCCCGACTTTGGCA
2	REN169O18	CACCCAACCTGTCTGTTCCT	ACTGTGTGAGCCAATCCCTT
3	REN54P11	GGGGGAATTAACAAAGCCTGAG	GTTTCTTTGCAAATTCTGAGCCCCACTG
4	INRA21	ATGTAGTTGAGATTTCTCCTACGG	GTTTCTTTAATGGCTGATTTATTTGGTGG
5	REN169D01	AGTGGGTTTGCAAGTGGAAC	AATAGCACATCTTCCCCACG
6	AHTh260	CGCTATACCCACACCAGGAC	GTTTCTTCCACAGAGGAAGGGATGC
7	INU005	CATGCTGGTTCTGTGTTAGGC	AAATACAATCTTGCGTGTGTGC
8	INU030	GGCTCCATGCTCAAGTCTGT	CATTGAAAGGGAATGCTGGT
9	AHT121	TATTGCGAATGTCACTGCTT	ATAGATACACTCTCTCTCCG
10	FH2054	GCCTTATTCATTGCAGTTAGGG	ATGCTGAGTTTTGAACTTTCCC
11	AHTh171	AGGTGCAGAGCACTCACTCA	GTTTCTTCCATCCACAGTTCAGCTTT
12	CXX279	TGCTCAATGAAATAAGCCAGG	GGCGACCTTCATTCTCTGAC
13	INU055	CCAGGCGTCCCTATCCATCT	GCACCACTTTGGGCTCCTTC
14	REN105L03	GGAATCAAAAGCTGGCTCTCT	GAGATTGCTGCCCTTTTTACC
15	AHTH137	TACAGAGCTCTTAACTGGGTCC	CCTTGCAAAGTGTCATTGCT
16	AHTH253	ACATTTGTGGGCATTGGGGCTG	GTTTCTTTGCACATGGAGGACAAGCACGC
17	AHTH130	GTTTCTCTCCCTTCGGGTTC	GACGTGTGTTCACGCCAG
18	FH2848	CAAAACCAACCCATTCACTC	TCACAAGGACTTTTCTCCTG
19	REN64E19	TGGAGAGATGATATCCAAAAGGA	AGCCACACTGCTTGGTGAG
20	REN162C04	TTCCCTTTGCTTTAGTAGGTTTTG	TGGCTGTATTCTTTGGCACA
21	REN2047M23	TGGTAACACCAAGGCTTTCC	TGTCTTTTCCATGGTGGTGA

**Table 2:** Polymorphism of 21 microsatellite DNA markers in the Polish Greyhound (n = 174).

*Locus*	Allele	Frequency of Alleles	PIC	Ho	He	FIS	*p*-Value Markov Chain Monte Carlo Test
AHTk211	87	0.057	0.477	0.580	0.570	−0.018	0.030 *
	89	0.445					
	91	0.477					
	95	0.020					
CXX279	116	0.178	0.695	0.764	0.731	−0.046	0.053
	118	0.431					
	120	0.037					
	124	0.055					
	126	0.193					
	130	0.106					
REN169O18	158	0.009	0.648	0.684	0.702	0.026	0.061
	162	0.233					
	164	0.293					
	166	0.032					
	168	0.040					
	170	0.394					
INU055	200	0.009	0.430	0.483	0.458	−0.055	0.010 **
	210	0.718					
	212	0.017					
	214	0.014					
	216	0.129					
	218	0.092					
	220	0.020					
REN54P11	226	0.239	0.667	0.707	0.715	0.011	0.616
	228	0.052					
	232	0.250					
	234	0.026					
	236	0.402					
	238	0.032					
INRA21	95	0.578	0.514	0.540	0.577	0.064	0.004 **
	97	0.017					
	101	0.124					
	103	0.282					
AHT137	131	0.580	0.560	0.638	0.602	−0.059	0.0.138
	133	0.221					
	137	0.035					
	143	0.023					
	147	0.098					
	153	0.043					
REN169d01	202	0.328	0.684	0.753	0.730	−0.031	0.003 **
	210	0.184					
	212	0.014					
	216	0.348					
	218	0.052					
	222	0.075					
AHTh260	240	0.023	0.696	0.759	0.738	−0.027	0.178
	244	0.072					
	246	0.175					
	248	0.351					
	252	0.313					
	254	0.066					
AHTk253	286	0.011	0.446	0.529	0.477	−0.108	0.869
	288	0.701					
	290	0.121					
	292	0.124					
	294	0.034					
	296	0.009					
INU005	110	0.161	0.474	0.477	0.513	0.070	0.121
	122	0.011					
	124	0.667					
	126	0.029					
	132	0.132					
INU030	144	0.422	0.432	0.580	0.535	−0.086	0.041 *
	148	0.009					
	150	0.534					
	152	0.034					
FH2848	234	0.043	0.651	0.701	0.705	0.006	0.113
	236	0.029					
	238	0.253					
	240	0.279					
	244	0.397					
AHT121	94	0.167	0.739	0.810	0.773	−0.048	0.044 *
	96	0.103					
	98	0.029					
	100	0.267					
	104	0.112					
	106	0.322					
FH2054	152	0.029	0.719	0.707	0.760	0.070	0.001 **
	156	0.305					
	160	0.210					
	164	0.181					
	168	0.264					
	172	0.011					
REN162c04	196	0.101	0.692	0.787	0.737	−0.068	0.004 **
	202	0.242					
	204	0.256					
	206	0.359					
	208	0.043					
AHT171	217	0.342	0.741	0.816	0.773	−0.056	0.028 *
	219	0.164					
	221	0.109					
	223	0.034					
	225	0.259					
	227	0.026					
	229	0.011					
	233	0.055					
REN247M23	266	0.247	0.476	0.529	0.532	0.005	0.062
	268	0.632					
	270	0.046					
	278	0.075					
REN64e19	139	0.170	0.767	0.764	0.798	0.042	0.028 *
	141	0.158					
	145	0.158					
	147	0.221					
	149	0.006					
	151	0.014					
	155	0.273					
AHTh130	121	0.091	0.361	0.374	0.384	0.026	0.013 *
	125	0.032					
	127	0.773					
	131	0.112					
REN115lo3	227	0.187	0.678	0.793	0.725	−0.094	0.418
	229	0.092					
	233	0.322					
	235	0.356					
	239	0.017					
	241	0.026					
$\bar{X}$			0.597	0.656	0.644	−0.018	

PIC—polymorphic information content. H_E_—expected heterozygosity. FIS—coefficient of inbreeding. HWE test: * *p* < 0.05; ** *p* < 0.01.

## Data Availability

The authors assure that their research complies with commonly accepted 3R principles. The tests (cheek swab) were non-invasive and were performed as part of a veterinary practice. The animals did not suffer any harm.
